# Modulation of Auditory Responses to Speech vs. Nonspeech Stimuli during Speech Movement Planning

**DOI:** 10.3389/fnhum.2016.00234

**Published:** 2016-05-18

**Authors:** Ayoub Daliri, Ludo Max

**Affiliations:** ^1^Speech Lab, Department of Speech, Language and Hearing Sciences, Boston UniversityBoston, MA, USA; ^2^Laboratory for Speech Physiology and Motor Control, Department of Speech and Hearing Sciences, University of WashingtonSeattle, WA, USA; ^3^Haskins LaboratoriesNew Haven, CT, USA

**Keywords:** speech, speech planning, auditory modulation, auditory evoked potentials, EEG/ERP

## Abstract

Previously, we showed that the N100 amplitude in long latency auditory evoked potentials (LLAEPs) elicited by pure tone probe stimuli is modulated when the stimuli are delivered during speech movement planning as compared with no-speaking control conditions. Given that we probed the auditory system only with pure tones, it remained unknown whether the nature and magnitude of this pre-speech auditory modulation depends on the type of auditory stimulus. Thus, here, we asked whether the effect of speech movement planning on auditory processing varies depending on the type of auditory stimulus. In an experiment with nine adult subjects, we recorded LLAEPs that were elicited by either pure tones or speech syllables when these stimuli were presented prior to speech onset in a delayed-response speaking condition vs. a silent reading control condition. Results showed no statistically significant difference in pre-speech modulation of the N100 amplitude (early stages of auditory processing) for the speech stimuli as compared with the nonspeech stimuli. However, the amplitude of the P200 component (later stages of auditory processing) showed a statistically significant pre-speech modulation that was specific to the speech stimuli only. Hence, the overall results from this study indicate that, immediately prior to speech onset, modulation of the auditory system has a general effect on early processing stages but a speech-specific effect on later processing stages. This finding is consistent with the hypothesis that pre-speech auditory modulation may play a role in priming the auditory system for its role in monitoring auditory feedback during speech production.

## Introduction

The central nervous system (CNS) modulates its response to sensory inputs that are consequences of self-produced movements. Studies have used behavioral and neurophysiological measures to examine this modulation in different sensory modalities (Waszak et al., 2012; Schröger et al., 2015b). Behavioral studies, for example, have shown that we perceive the loudness of a sound that is a consequence of our own action as less intense than the loudness of a sound produced by others (Sato, 2008, 2009; Weiss et al., 2011; Desantis et al., 2012). Using neurophysiological techniques—such as electroencephalography (EEG), magnetoencephalography (MEG), electrocorticography (ECoG), and single unit recordings—studies have shown that cortical responses evoked by self-produced speech sounds are modulated in comparison with those evoked by hearing a played-back version of the same speech sounds^1^ (EEG: Ford et al., 2001; Liotti et al., 2010; MEG: Curio et al., 2000; Houde et al., 2002; Beal et al., 2010; ECoG: Towle et al., 2008; Greenlee et al., 2011; single unit recordings: Creutzfeldt et al., 1989). The mechanism underlying this modulation is precise and specific: experimentally implemented or naturally occurring deviations in the feedback signal result in a reduction of the modulation magnitude (Heinks-Maldonado et al., 2006; Chang et al., 2013; Niziolek et al., 2013). In addition, animal studies have further confirmed this phenomenon in the auditory-motor system of monkeys, rodents, bats, and crickets (Suga and Schlegel, 1972; Suga and Shimozawa, 1974; Muller-Preuss and Ploog, 1981; Poulet and Hedwig, 2002, 2007; Eliades and Wang, 2003; Nelson et al., 2013; Schneider et al., 2014).

Typically, these results have been interpreted in the context of theoretical frameworks of motor control involving efference copy and forward internal models (Waszak et al., 2012; Horvath et al., 2015; Schröger et al., 2015b). Specifically, it has been suggested that the CNS uses an efference copy of issued motor commands and forward internal models to predict the auditory consequences of self-produced movements. The CNS then compares this prediction with the actual auditory feedback, and it attenuates its response to auditory feedback that matches the prediction (Houde et al., 2002; Heinks-Maldonado et al., 2006; Behroozmand and Larson, 2011; Chang et al., 2013; Niziolek et al., 2013).

In addition to such sensory modulation during movement execution, a growing body of evidence suggests that the CNS already modulates sensory processing during movement planning (Creutzfeldt et al., 1989; Max et al., 2008; Mock et al., 2011, 2015; Daliri and Max, 2015a,b). In a recent speech study (Daliri and Max, 2015b), we recorded long latency auditory evoked potentials (LLAEPs) in response to probe tones that were played either prior to speaking (i.e., during speech movement planning) or during no-speaking control conditions (silent reading or seeing nonlinguistic symbols). Results for a group of participants with typical speech showed that N100 amplitude in the speaking condition was attenuated in comparison with both control conditions. We suggested that, during speech planning, the CNS uses an efference copy of planned control signals to prime the auditory system for its role in processing the upcoming auditory feedback resulting from execution of those control signals. In our previous studies (Daliri and Max, 2015a,b), we examined pre-speech auditory modulation only by means of pure tone probe stimuli. Thus, it has remained unknown whether the overall nature and magnitude of this auditory modulation vary for different types of stimuli. To elucidate the phenomenon’s potential role in the monitoring of auditory feedback during speech production, however, it is necessary to first understand whether speech movement planning differentially affects the auditory system’s processing of speech stimuli as compared with nonspeech stimuli. Hence, in the present study, we aimed to investigate whether the effect of speech movement planning on auditory processing varies depending on the general characteristics of the auditory probe stimulus. Specifically, we studied pre-speech modulation of LLAEPs that were elicited by either pure tones or speech syllables. For both types of stimuli, we quantified pre-speech auditory modulation by comparing the N100 and P200 components’ amplitudes in a speaking condition vs. a silent reading condition.

N100 is the largest negative peak in the electrical cortical response to a transient auditory stimulus, occurring approximately 100 ms after onset of the stimulus. The N100 component is primarily generated by neural populations located in the primary auditory cortex (Näätänen and Picton, 1987; Zouridakis et al., 1998; Godey et al., 2001) and reflects processes involved in detecting acoustic change in the environment (e.g., Hyde, 1997). P200 is the largest positive peak that follows N100, approximately 180 ms after onset of the auditory stimulus. The neural generators of P200 are less well understood. It has been suggested that primary neural generators of P200 are located in multiple auditory areas, including primary and secondary auditory cortices (Hari et al., 1987; Scherg et al., 1989; Baumann et al., 1990; Mäkelä and Hari, 1990; Godey et al., 2001; Steinschneider and Dunn, 2002). However, it has also been suggested that P200 may have additional neural generators separate from the auditory areas (Crowley and Colrain, 2004). The functional role of the P200 component is not entirely clear (Crowley and Colrain, 2004). For speech stimuli, however, the available data suggest that whereas the N100 component is involved in early stages of auditory processing such as encoding basic physical aspects of the auditory input, the P200 component is involved in later stages of auditory processing such speech-specific, higher-level analysis (Näätänen and Picton, 1987; Crowley and Colrain, 2004; Tremblay et al., 2009; Pratt and Lightfoot, 2012). Therefore, in the current study, we hypothesized that if pre-speech modulation plays a role in priming the auditory system for its role in monitoring auditory feedback during speech production, then the N100 and P200 components might be differentially affected when the stimuli used to probe the auditory system during speech planning are pure tones vs. speech syllables.

## Materials and Methods

### Participants

Participants were nine right-handed adults (3 females; age range: 22–30 years, *M* = 25.44 years, *SD* = 2.45) who were naive to the purpose of the study. All participants were native speakers of American English who self-reported no current or prior neurological, psychological, or communication disorders. Only participants with normal binaural hearing (≤20 dB hearing level (HL) at octave frequencies 250–8000 Hz) were included. Prior to the experiment, written informed consent was obtained from all subjects. All procedures were approved by the Institutional Review Boards of the University of Washington (where the data were collected and analyzed).

### Instrumentation and Procedure

The design of this study was based on our previously published studies (Daliri and Max, 2015a,b). During the test session, each participant was seated inside a sound-attenuated room in front of a 23-inch LCD monitor. The participant’s speech output was transduced with a wireless microphone (WL185, Shure Incorporated, Niles, IL, USA) placed approximately 15 cm from the mouth, amplified with a microphone amplifier (DPS II, ART ProAudio, Niagara Falls, NY, USA) and headphone amplifier (S.phone, Samson Technologies Corp., Syosset, NY, USA), and played back to the participant through insert earphones (ER-3A, Etymotic Research Inc., Grove Village, IL, USA). Prior to each experiment, the overall amplification level was calibrated such that a 75 dB SPL speech signal at the microphone produced an output of 73 dB sound pressure level (SPL) in the insert earphones. The earphones’ output level was measured with a 2 cc coupler (Type 4946, Bruel and Kjaer Inc., Norcross, GA, USA) connected to a sound level meter (Type 2250A Hand Held Analyzer with Type 4947 $\frac{1}{2}$” Pressure Field Microphone, Bruel and Kjaer Inc., Norcross, GA, USA).

**Figure 1:**
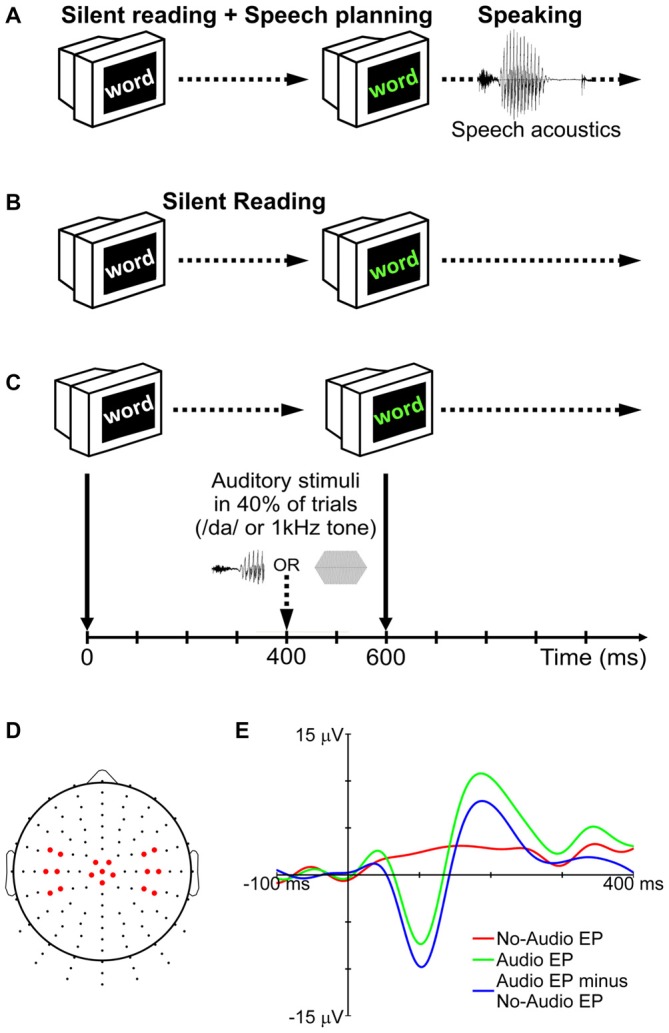
**Experimental procedure for the speaking (A) and silent reading (B) conditions.** In 40% of trials (audio trials), auditory stimuli (either the syllable /da/ or a 1 kHz tone) were presented during the delay period **(C)**. No auditory stimuli were delivered in the remaining trials (no-audio trials). To remove the effect of non-auditory processes (motor, visual, linguistic, etc.) from the long-latency auditory evoked potentials (LLAEPs), evoked potentials (EPs) of no-audio trials were subtracted from EPs of audio trials **(E)**. The final LLAEPs in three regions of interest (ROIs) were entered into the statistical analyses **(D)**. Figure is adapted and updated from Daliri and Max (2015b), Page 61, Copyright © 2015, with permission from Elsevier.

Participants completed five blocks of trials for a speaking condition and five blocks of trials for a silent reading condition (Figures 1A,B). The order of the 10 blocks was randomized for each participant. Prior to the start of each block, participants were informed about the condition to be completed in that block (note that each block contained trials for only one condition). Each block consisted of 90 trials. In each trial, a word in white characters was presented against a black background on the computer monitor (Figure 1C). The color of the word changed to green after 600 ms. In the speaking condition, this change of color signaled the *go* cue for the participant to say the word aloud. Participants ignored the characters’ change of color in the silent reading condition. During 40% of the trials in each block (audio trials), auditory stimuli were delivered binaurally through the insert earphones. In the remaining trials (no-audio trials), no auditory stimuli were presented.

The auditory stimulus was either a pure tone (1 kHz; 40 ms duration; 10 ms rise/fall time; 75 dB SPL) or a truncated recording of the syllable /da/ (40 ms duration; 75 dB SPL) spoken by the participant at the beginning of the session. Praat 5.3 (Boersma and Weenink, 2016) was used to record the audio signal from the production of the syllable, and to truncate it to the first 40 ms. The rationale for truncation was to keep the length of the two stimuli the same (tone and syllable), and to maintain consistency with our previous studies (Daliri and Max, 2015a,b). As shown in Figure 1C, auditory stimuli were presented 400 ms after appearance of the target word in white characters (for a discussion on the time-point of stimulus delivery, see Max et al., 2008). The trial ended when the word disappeared from the screen 500 ms after the *go* signal. The temporal interval between two successive trials was randomly selected from a set of possible intervals (1500, 2000, 2500, 3000, or 3500 ms). The words to be spoken or read in each block of trials were randomly selected from a list containing 300 monosyllabic consonant-vowel-consonant (CVC) words with no consonant clusters. All words were 3–5 letters long. Psyscope X B53 was used: (a) to present words on the screen; (b) to deliver auditory stimuli to participants; and (c) to send external triggers for the EEG system (see below).

### Electroencephalographic Recordings

Using a Biosemi active-electrode EEG system with Ag/AgCl electrodes (Active Two, Biosemi Inc., Amsterdam, Netherlands), EEG signals were recorded from 128 standard sites on the scalp (Figure 1D) according to an extension of the international 10–10 electrode system (Gilmore, 1994; Oostenveld and Praamstra, 2001). Electrooculogram (EOG) signals related to blinking and eye movements were recorded using two electrodes placed on the outer canthus and below the left eye. Electromyogram (EMG) signals related to orofacial muscle activity were recorded using four electrodes placed on the skin overlying right-side orofacial muscles (masseter: jaw elevation; anterior belly of the digastric: jaw depression; orbicularis oris: lower and upper lip displacement/rounding). Serving as reference electrodes, two additional electrodes were placed on the left and right mastoids. The signals from all electrodes (EEG, EOG, EMG, references) and from an additional microphone placed 15 cm away from the participant’s mouth (SM58, Shure, Niles, IL, USA) were continuously recorded at a sampling rate of 1024 Hz.

### Data Analysis

The EEGLAB toolbox (Delorme and Makeig, 2004) and custom-written MATLAB scripts (The MathWorks, Inc., Natick, MA, USA) were used for offline data analysis. Signals from the two mastoid electrodes were mathematically averaged to reconstruct a reference signal. All EEG signals were re-referenced to this average mastoid signal and low-pass filtered with a cut-off frequency of 50 Hz using a finite impulse response (FIR) filter (Kaiser windowed sinc FIR filter; deviation: 0.005; transition bandwidth: 1 Hz). The continuous data were then segmented into epochs from 100 ms before the auditory stimulus to 400 ms after the auditory stimulus in audio trials or the equivalent time interval in no-audio trials. To adjust for baseline differences across epochs, the average amplitude of the 100 ms pre-stimulus period for each epoch was subtracted from the whole epoch. Epochs were inspected to reject those with: (a) EEG amplitudes exceeding ±100 μV; (b) large EOG signals associated with eye movements and blinking; and (c) EMG activity before the *go* signal. Each participant’s remaining epochs for the audio trials and no-audio trials from a given condition (either speaking or silent reading) were then averaged separately.

A participant’s averaged response for audio trials (tone or syllable) reflected brain activity related to both auditory and non-auditory processing (e.g., activity associated with motor, linguistic, cognitive, and visual processes necessary to complete the task). The averaged response for no-audio trials, on the other hand, reflected only brain activity related to the non-auditory processes. Therefore, to derive LLAEPs that best estimated the actual auditory response, each participant’s averaged response for no-audio trials was subtracted from her or his averaged response for audio trials (Martikainen et al., 2005; Bäess et al., 2008, 2011; Luck, 2014; Daliri and Max, 2015a,b). Figure 1E illustrates this procedure which was used to derive LLAEPs for all individual electrodes.

As a last step, directly motivated by the results from our prior work (Daliri and Max, 2015a,b), the data from selected electrodes located in three regions of interest (ROIs) were averaged. As illustrated in Figure 1D, these ROIs included electrodes over the left hemisphere (Left ROI: electrodes D11, D12, D19 [equivalent to C3], D20, D27, D28), the central region (Central ROI: electrodes A1 [equivalent to Cz], A2, B1, C1, D1, D15), and the right hemisphere (Right ROI: electrodes B17, B18, B22 [equivalent to C4], B23, B30, B31). Our previous work (Daliri and Max, 2015b) indicated that auditory modulation is larger at electrodes located over the central region and over the left hemisphere than over the right hemisphere. Thus, we used the same ROIs for the present study as this allows an examination of the consistency of such ROI effects, if any, on the auditory modulation phenomenon. The evoked response obtained for each ROI was further low-pass filtered (cut-off frequency 15 Hz; Kaiser windowed sinc FIR filter; deviation: 0.005; transition bandwidth: 1 Hz) before the peak amplitude and peak latency of the N100 and P200 components were extracted. N100 was defined as the largest negative peak between 70 and 130 ms, and P200 was defined as the largest positive peak between 150 and 250 ms. We used a custom written MATLAB script together with visual verification to detect N100 and P200 peaks for each individual participant in each of the conditions (Luck, 2014).

### Statistical Analyses

IBM SPSS Statistics 19 (IBM, Armonk, NY, USA) was used to conduct the statistical analysis. For each dependent variable, analysis of variance (ANOVA) for repeated measures was used with Condition (speaking and silent reading), Stimulus (tone and syllable), and ROI (left ROI, central ROI, and right ROI) as the repeated measures. To account for potential violations of the sphericity assumption, degrees of freedom were adjusted using the Huynh-Feldt correction (Max and Onghena, 1999). As appropriate, repeated measures of ANOVA were followed up by *post hoc* analyses using *t*-tests with Bonferroni corrections for multiple comparisons.

## Results

**Figure 2:**
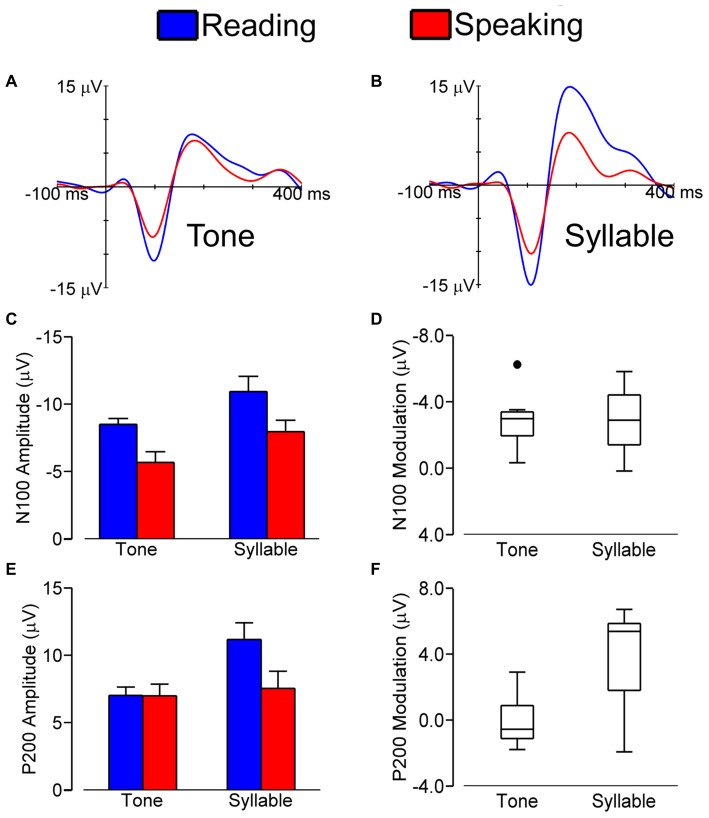
**Grand average (over all subjects) LLAEPs in response to tones (A) and syllables (B) for the central ROI in the speaking (red) and the silent reading (blue) conditions.** Bar graphs show the group average N100 amplitude **(C)** and P200 amplitude **(E)**, with error bars indicating standard errors. Each bar represents data averaged across participants and ROIs. Box plots show the distribution of N100 modulation **(D)** and P200 modulation **(F)**. We found similar N100 amplitude modulation for tones and syllables. For P200 amplitude on the other hand, statistically significant modulation was found for responses elicited by syllables and not for responses elicited by tones.

Figure 2 shows grand average LLAEP (averaged over all subjects) waveforms from the central ROI when tones (A) or syllables (B) were presented during speech planning and during silent reading. As illustrated, the LLAEP amplitudes for both tones and syllables are reduced in the speaking condition as compared with the silent reading condition. Statistical results for measures of N100 and P200 amplitude and latency are described in the following sections.

### N100 Amplitude

Figure 2C shows N100 amplitudes in the speaking and reading conditions for both tones and syllables. The magnitude of N100 amplitude modulation (i.e., amplitude in the reading condition minus amplitude in the speaking condition) for tones vs. syllables is shown in Figure 2D.

We found a statistically significant main effect of Stimulus, *F*_(1,8)_ = 13.927, *p* = 0.006, with a larger N100 amplitude in response to syllables vs. tones. We also found statistically significant main effects of Condition, *F*_(1,8)_ = 35.601, *p* < 0.001, and ROI, *F*_(1.562,12.498)_ = 57.199, *p* < 0.001, as well as a Condition × ROI interaction, *F*_(2,16)_ = 9.602, *p* = 0.002. This interaction occurred because the magnitude of N100 modulation (i.e., the difference in N100 amplitude between the speaking and reading conditions) was larger in the central ROI than in the left ROI, *t*_(8)_ = −3.401, *p* = 0.009, or the right ROI, *t*_(8)_ = −4.148, *p* = 0.003. Most relevant to the hypothesis under investigation, however, is that the Stimulus × Condition interaction was not statistically significant (*p* = 0.841). Thus, with regard to N100 amplitude, pre-speech auditory modulation did not differ for speech vs. non-speech stimuli. Lastly, we also found no statistically significant interactions of Stimulus × ROI (*p* = 0.074) or Condition × Stimulus × ROI (*p* = 0.097).

### N100 Latency

N100 latency data showed a statistically significant main effect of Stimulus, *F*_(1,8)_ = 35.051, *p* < 0.001, and a statistically significant Stimulus × Condition interaction, *F*_(1,8)_ = 5.450, *p* = 0.048. The N100 latency was longer for responses to syllables than responses to tones (110.7 ms vs. 98.2 ms), and the difference between N100 latencies for syllables vs. tones was larger in the speaking condition than in the reading condition (15.7 ms vs. 9.3 ms). None of the other main effects (Condition and ROI) or interactions (Condition × ROI, Stimulus × ROI, and Condition × Stimulus × ROI) were statistically significant (*p* > 0.069 in all cases).

### P200 Amplitude

Figure 2E illustrates P200 amplitudes in both conditions (speaking and reading) and in response to both stimuli (tone and syllable); the magnitudes of the modulation of P200 amplitudes in response to tones and syllables are shown in Figure 2F.

P200 amplitude data showed statistically significant main effects of Condition, *F*_(1,8)_ = 7.884, *p* = 0.023, Stimulus, *F*_(1,8)_ = 7.938, *p* = 0.023, and ROI, *F*_(1.460,11.681)_ = 30.675, *p* < 0.001. These effects were modified by statistically significant interactions of Condition × ROI, *F*_(1.572,12.218)_ = 19.917, *p* < 0.001, Stimulus × ROI, *F*_(2,16)_ = 6.684, *p* = 0.008, Condition × Stimulus, *F*_(1,8)_ = 11.834, *p* = 0.008, and Condition × Stimulus × ROI, *F*_(1.438,11.507)_ = 4.579, *p* = 0.044. Most important for the aim of the present study, these results showed that only for the auditory responses to syllables—and not those to tones—the P200 amplitude in the speaking condition was statistically significantly smaller than the P200 amplitude in the reading condition, *t*_(8)_ = 3.460, *p* = 0.008. This difference (i.e., P200 modulation, defined as P200 amplitude in the reading condition minus P200 amplitude in the speaking condition) was larger in the central ROI than in the left ROI, *t*_(8)_ = 3.816, *p* = 0.005, and the right ROI, *t*_(8)_ = 4.863, *p* = 0.001.

### P200 Latency

P200 latency data showed no statistically significant main effects of Stimulus (*p* = 0.750), Condition (*p* = 0.500), or ROI (*p* = 0.169). Furthermore, none of the two-way and three-way interactions were statistically significant (*p* > 0.086 in all cases).

## Discussion

We previously showed that, during speech planning, the CNS modulates auditory responses to nonspeech, pure tone probe stimuli (Daliri and Max, 2015a,b). We suggested that, during the preparation of speech movements, the CNS uses an efference copy of planned control signals to prime the auditory system for its role in processing the upcoming auditory feedback resulting from execution of those control signals (Daliri and Max, 2015a,b). However, to elucidate this auditory modulation phenomenon’s potential role in the monitoring of auditory feedback, it is essential to understand whether speech movement planning differentially affects the auditory system’s processing of speech stimuli as compared with nonspeech stimuli. Thus, in the present study, we investigated whether the effect of speech movement planning on auditory processing varies depending on the general characteristics of the auditory probe stimulus. We studied pre-speech modulation of LLAEPs elicited by self-produced, pre-recorded syllables vs. pure tones when these stimuli were presented during speech planning or silent reading. We hypothesized that if pre-speech modulation plays a role in priming the auditory system for its role in monitoring auditory feedback during speech production, then the N100 and P200 components might be differentially affected for pure tone probes as compared with speech probes.

With direct relevance to this main hypothesis, we report three primary findings. First, we replicated again (see also Daliri and Max, 2015a,b) a statistically significant modulation of the auditory N100 amplitude during speech movement planning in comparison with silent reading. Second, as an entirely novel result, we found that the magnitude of this N100 amplitude modulation did not differ for responses evoked by the speech vs. nonspeech stimuli used here. Third, we also found a statistically significant modulation of the auditory P200 amplitude, but this P200 modulation was exclusive to responses evoked by speech stimuli and did not occur for nonspeech stimuli. Thus, the overall results from this study indicate that immediately prior to speech onset, modulation of the auditory system has: (a) a general effect on early auditory processing stages (as evident by similar magnitudes of N100 modulation for responses elicited by speech and nonspeech stimuli); but (b) a speech-specific effect on later processing stages (as evident by significant modulation of P200 amplitude for response elicited by speech stimuli but not nonspeech stimuli). This pattern of results is consistent with the proposed hypothesis that pre-speech auditory modulation may play a role in priming the auditory system for its role in monitoring auditory feedback during speech production.

Although this study cannot answer the question why the modulating influence of speech planning is general during the early stages of auditory processing (100 ms after stimulus onset) but speech-specific during later stages of auditory processing (200 ms after stimulus onset), we offer two possible, although not necessarily mutually exclusive, explanations. The first explanation is based on differences in the role of such separate stages of auditory processing. It has been suggested previously that, for speech stimuli, early stages of auditory processing encode basic physical aspects of the auditory input whereas later stages are involved in speech-specific, higher-level processing (Näätänen and Picton, 1987; Crowley and Colrain, 2004; Tremblay et al., 2009; Pratt and Lightfoot, 2012). If, during speech planning, the CNS already prepares the auditory system for its role in processing upcoming auditory feedback, it may modulate neuronal populations involved in processing the basic physical properties of any acoustic input as well as neuronal populations only involved in higher levels of processing speech-specific information. Thus, probing the auditory system during the speech planning stage with stimuli that do not have speech-like characteristics (such as our pure tone stimulus) may reveal only the modulation of the former population of neurons (resulting in decreased N100 amplitude prior to speaking) whereas probing with speech stimuli (such as our syllable stimulus) may reveal the modulation of both populations of neurons (resulting in decreases in both N100 and P200 amplitude prior to speaking).

The second proposed explanation relates to the time course of auditory processing. Given that the LLAEP P200 component occurs approximately 100 ms after the N100 component, our P200 measurements are extracted at a time point closer to movement onset. If the CNS incrementally refines its motor commands during the movement planning stage, its modulating signals to auditory cortex may—in parallel—also become more specific over time. Thus, in comparison with the neural generators of the N100 component, the neural generators of the P200 component may receive modulating signals that carry more refined information about the expected input. As we have suggested previously (Daliri and Max, 2015a,b), this hypothesis can be tested empirically by examining pre-speech auditory modulation at different time points relative to movement onset.

Some authors have suggested that the phenomenon of auditory modulation may reflect general attentional processes rather than motor-to-auditory processes (see Jones et al., 2013; Horvath et al., 2015; Schröger et al., 2015a). If so, it could be argued that our present paradigm’s use of a delayed-response task (i.e., participants actively withhold a planned utterance until the *go* signal is presented) reduces auditory attention in the speaking task. As a result of such reduced attention allocation, the LLAEP amplitudes could also be reduced. However, the findings from other studies as well as aspects of our own overall methodology make an attention-based interpretation of the present results highly unlikely. First, several studies have shown that experimentally manipulating the allocation of attention (i.e., attending to the auditory stimuli vs. attending to the motor task vs. attending to unrelated visual stimuli) does not influence the magnitude of auditory modulation, at least not during movement execution (SanMiguel et al., 2013; Saupe et al., 2013; Timm et al., 2013). Second, our most important result relates to a difference in pre-speech auditory modulation for pure tone vs. speech stimuli. These two types of stimuli were presented in randomized (i.e., non-predictable) order within the trial blocks for both the speaking condition and the silent reading condition. When a participant was planning and withholding a speech response, no cues were available to indicate whether a pure tone or a speech syllable (or no auditory stimulus at all) would be heard during that trial. Thus, auditory attention would have affected the responses to the different stimuli in similar ways.

Lastly, one potential caveat related to our auditory stimuli should be acknowledged. Although the two stimuli had the same duration, intensity, and rate of presentation, there were differences in several physical characteristics, including the rise/fall time, maximum amplitude, temporal envelope and spectral complexity. It could be argued that the differential modulation of auditory processing that is reflected in the P200 component may be a result of, or influenced by, such basic stimulus characteristics. The methodology used here cannot rule out this possibility. Future research along these lines should include studies examining the effects on auditory modulation of stimuli that differ in only one of these characteristics.

In summary, we found a statistically significant modulation of auditory N100 amplitude when either speech or nonspeech stimuli were presented prior to speaking vs. silent reading, and the magnitude of this modulation was similar for both types of stimuli. However, we also found that statistically significant modulation of the P200 amplitude was specific for speech stimuli and did not occur with pure tone stimuli. Together, these results may indicate that, immediately prior to speech onset, modulation of the auditory system has a general effect on early processing stages but a speech-specific effect on later processing stages. This finding is consistent with the hypothesis that pre-speech auditory modulation may play a role in priming the auditory system for its role in monitoring auditory feedback during speech production.

## Author Contributions

AD and LM designed and conducted the experiment. AD analyzed the data. AD and LM interpreted the results and wrote the manuscript.

## Conflict of Interest Statement

The authors declare that the research was conducted in the absence of any commercial or financial relationships that could be construed as a potential conflict of interest.

## Footnotes

^1^Similar results have been obtained when participants press a button that causes a tone to be played vs. passively listening to tones (for a review see Schröger et al., 2015b).
